# Effects of municipal smoke-free ordinances on secondhand smoke exposure in the Republic of Korea

**DOI:** 10.3389/fpubh.2023.1062753

**Published:** 2023-03-27

**Authors:** Siwoo Kim, Yuri Lee, Changwoo Han, Min Kyung Kim, Ichiro Kawachi, Juhwan Oh

**Affiliations:** ^1^Institute of Environmental Medicine, SNU Medical Research Center, Seoul National University, Seoul, Republic of Korea; ^2^Department of Health and Medical Information, Myongji College, Seoul, Republic of Korea; ^3^Department of Preventive Medicine, Chungnam National University College of Medicine, Daejeon, Republic of Korea; ^4^Tufts Clinical and Translational Science Institute, Boston, MA, United States; ^5^Department of Social and Behavioral Sciences, Harvard T. H. Chan School of Public Health, Boston, MA, United States; ^6^Department of Medicine, Seoul National University College of Medicine, Seoul, Republic of Korea

**Keywords:** tobacco control, secondhand smoke, smoke-free policies, municipal smoke-free ordinances, interrupted time series analysis, health policy impact

## Abstract

**Objective:**

To reduce premature deaths due to secondhand smoke (SHS) exposure among non-smokers, the Republic of Korea (ROK) adopted changes to the National Health Promotion Act, which allowed local governments to enact municipal ordinances to strengthen their authority to designate smoke-free areas and levy penalty fines. In this study, we examined national trends in SHS exposure after the introduction of these municipal ordinances at the city level in 2010.

**Methods:**

We used interrupted time series analysis to assess whether the trends of SHS exposure in the workplace and at home, and the primary cigarette smoking rate changed following the policy adjustment in the national legislation in ROK. Population-standardized data for selected variables were retrieved from a nationally representative survey dataset and used to study the policy action’s effectiveness.

**Results:**

Following the change in the legislation, SHS exposure in the workplace reversed course from an increasing (18% per year) trend prior to the introduction of these smoke-free ordinances to a decreasing (−10% per year) trend after adoption and enforcement of these laws (*β*_2_ = 0.18, *p*-value = 0.07; *β*_3_ = −0.10, *p*-value = 0.02). SHS exposure at home (*β*_2_ = 0.10, *p*-value = 0.09; *β*_3_ = −0.03, *p*-value = 0.14) and the primary cigarette smoking rate (*β*_2_ = 0.03, *p*-value = 0.10; *β*_3_ = 0.008, *p*-value = 0.15) showed no significant changes in the sampled period. Although analyses stratified by sex showed that the allowance of municipal ordinances resulted in reduced SHS exposure in the workplace for both males and females, they did not affect the primary cigarette smoking rate as much, especially among females.

**Conclusion:**

Strengthening the role of local governments by giving them the authority to enact and enforce penalties on SHS exposure violation helped ROK to reduce SHS exposure in the workplace. However, smoking behaviors and related activities seemed to shift to less restrictive areas such as on the streets and in apartment hallways, negating some of the effects due to these ordinances. Future studies should investigate how smoke-free policies beyond public places can further reduce the SHS exposure in ROK.

## 1. Introduction

Tobacco epidemic is a global public health issue (1). There is no safe level of tobacco smoke, and even brief exposure to it can cause serious harm (2). Annually, more than 1.2 million people die from indirect exposure to tobacco smoke (3). Secondhand smoke (SHS) exposure, as it is called, remains a significant cause of respiratory illnesses, cardiovascular diseases, cancer, and premature deaths among non-smokers (2, 4). Acknowledging this need to protect non-smokers from inhaling tobacco smoke, Article 8.2 of the World Health Organization Framework Convention for Tobacco Control (WHO FCTC) recommends that all countries implement effective legislative, executive, administrative, and other policy measures to protect people from tobacco smoke in indoor workplaces, public transport, indoor public places, and other public places (5, 6). As such, member countries who signed on to the WHO FCTC became responsible for adopting smoke-free legislation in their jurisdictions.

Previous studies have reported that smoke-free legislations are effective in reducing the social acceptability of smoking, fostering an environment for smoking regulation, limiting opportunities to smoke, and creating smoke-free environments across affected areas (6–8). In the United Kingdom, the Smoke-Free Premises and Enforcement Regulation sets designated smoke-free area where local councils and port health authorities are responsible for enforcing a fixed penalty of 30–200 Pound(£) for smoking (9). This smoke-free legislation was effective in reducing SHS exposure and improving the cardiovascular health of residents in the United Kingdom (10). In Japan, despite the enactment of the Health Promotion Act for the prevention of lifestyle-related diseases in 2003, only a few regulatory elements supported smoking restriction in public places. Local governments eventually stepped in and played a significant role in promoting smoke-free environments in Japan by promoting municipal ordinances to restrict smoking in indoor public places and on the streets, and enforcing penalties for violators of these laws (11). Among the lessons learned was that simple enactment or adoption of legislation often fails to change people’s smoking behaviors or motivate them to comply with legislation (12–14). Penalties for violations alongside other nudges are required to encourage people’s participation (15, 16).

In the Republic of Korea (ROK), 32.3 and 5.3% of male and female deaths, respectively, were attributed to smoking in 2019. The economic burden due to direct and indirect exposure to tobacco smoke has been estimated to be 12 trillion Korean Won (KRW) in 2019 (17). The smoke-free policies in ROK became law in 1995 with the establishment of the National Health Promotion Act. Since then, various smoke-free policies and legislations have followed. Smoke-free areas were designated in indoor public facilities, large buildings, concert halls, wedding halls, in-door gymnasiums, health facilities, social welfare facilities, etc. (18). However, the lack of penalties for smoking in non-smoking zones and weak enforcement powers of local governments made it difficult to protect non-smokers from SHS exposure. In May 2010, the introduction of municipal ordinances under the National Health Promotion Act eventually allowed local governments to exercise autonomous legislative powers by laying down local ordinances, designating outdoor public places as non-smoking areas, and enforcing fines for individuals caught smoking in non-smoking zones (19, 20). By 2012, about 51% of local governments had implemented smoke-free ordinances, and 41% of them initiated the ordinances for more than 1 year (21). The local governments started to designate non-smoking areas such as streets and bus stops while enforcing the fines (from 20,000 KRW to 100,000 KRW) for smoking in non-smoking areas (22).

Although many studies conducted in ROK have assessed the effects of smoke-free policies, little is known about whether the introduction of municipal smoke-free ordinances under the national legislation affected the SHS exposure trends. Previously, Park et al. (23) analyzed the concentration of cotinine levels in urine among non-smoking workers before and after the introduction of municipal ordinances from 2009 to 2011. Whereas the urine cotinine concentration continued to decrease during the study period, the study only included 2,475 non-smoking workers (23). No studies to date have evaluated longitudinal changes in SHS exposure using nationally representative data. Furthermore, no study has compared SHS exposure in the workplace and at home since the introduction of ordinances under the national legislation in 2010.

The implications for understanding the national-local policymaking dynamics are plentiful. For example, potential pathways through which smoke-free legislation affect SHS exposure include the following: first, policy-specific facilitators such as increasing public support for smoke-free legislation are often needed; second, psychosocial mediators such as attitudes, subjective norms, and self-efficacy, perceived risk, and perceived behavioral control increase or impede compliance with the law; third, psychosocial mediators lead to individual- and population-level outcomes such as quit attempts, smoking prevalence, and SHS exposure in public places (24). Therefore, we expected that allowing municipal ordinances under the national legislation would have certainly strengthened the role of local governments to better manage smoking in public places and protect non-smokers, for both male and female citizens of ROK (25).

In this study, we examined the effects of the change made in municipal ordinances under the national legislation in 2010 on SHS exposure among adults in ROK. First, we examined SHS exposure in the workplace and at home. Second, we conducted a subgroup analysis by sex.

## 2. Methods

### 2.1. Data sources

We used variables related to SHS exposure and smoking status from the nationally representative survey, Korea National Health and Nutrition Examination Survey (KNHANES).^1^ KNHANES is a nationwide health survey of adult population aged 19 and above conducted by the Korea Disease Control and Prevention Agency (KDCA) in ROK since 1998. Although the survey was conducted annually or biannually during 1998–2008, it has been fielded annually since 2010. Because the survey tool has been revised over time, data availability for each study variable varies. For example, while data for the primary cigarette (current) smoking rate were available from 1998 to 2020, the data for SHS exposure in the workplace and at home were only available from 2005 to 2020. These data are typically aggregated as proportions based on individual data—i.e., KNHANES provides data for proportions and population-standardized proportions based on individual data. For our study, we retrieved and analyzed data on overall, male, and female populations and population-standardized proportions from the KNHANES.

### 2.2. Measures

From the KNHANES, three variables—SHS exposure in the workplace, SHS exposure at home, and primary cigarette smoking rate—were used for the study. SHS exposure in the workplace was measured as percentage of non-smokers, including previous smokers, who were exposed to smoke indoors at work in the last 7 days. SHS exposure at home was measured as percentage of people who were exposed to smoke at home in the last 7 days. Primary cigarette smoking rate was measured as the number of people who reported smoking at least 100 cigarettes during their lifetime and reported smoking every day or some days when they participated in the survey.

### 2.3. Statistical analysis

We used interrupted time series analysis to assess whether the trends of the SHS exposure in workplace, at home, and the current smoking rate changed following the introduction of smoke-free municipal ordinances under the national legislation in 2010. Interrupted time series analysis is a powerful quasi-experimental research design, mainly used when there is no control group (27). It examines whether an intervention that took place at some point made any change in the post-intervention period, thus, provides statistical evidence of an intervention in action (28). For our study, we fit the following linear regression equation:

log(SHS exposure)*_y_* = *β*_0_ + *β*_1_⋯time*_y_* + *β*_2_⋯intervention*_y_* + *β*_3_⋯time after intervention*_y_* + e*_y_*.

log(current smoking rate*_y_*) = *β*_0_ + *β*_1_⋯time*_y_* + *β*_2_⋯intervention*_y_* + *β*_3_⋯time after intervention*_y_* + e*_y_*.

where *y* is the year; time*_y_* counts the number of years starting from the available data year; intervention*_y_* is the dummy variable when zero means the intervention is yet to be implemented and one means the intervention is implemented; and time after intervention*_y_* counts the number of years from 2010 when the smoke-free municipal ordinances under the national legislation (intervention) were first introduced. From the right side of the equation, *β*_0_ is the intercept, *β*_1_ is the baseline trend before the intervention, *β*_2_ is the very first impact of the intervention, and *β*_3_ is the trend change after the intervention.

We performed an original linear regression and then applied log transformation to the dependent variable. To test for autocorrelation, the Durbin–Watson statistic was used. We used R software (R x64 3.6.0) to perform these statistical analyses.

## 3. Results

### 3.1. Overall trends

**Figure 1:**
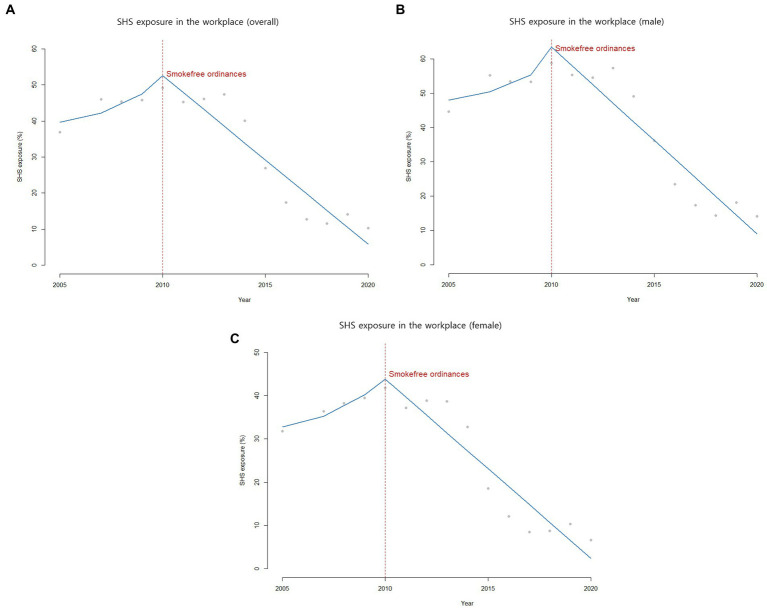
SHS exposure in the workplace among adults in ROK (2005-2020).

SHS exposure in the workplace showed an increasing trend from 36.9% in 2005 to 45.8% in 2009. Starting in 2010; however, SHS exposure in the workplace began to decrease from 49.2% in 2010 to 10.3% in 2020 (Table 1; Figure 1). Enforcement of 2010 municipal ordinances was not associated with an immediate change in the intercept for SHS exposure in the workplace. However, its slope fell by −10% annually after the new legislation adjustment took place (*β*_2_ = 0.18, *p*-value = 0.07; *β*_3_ = −0.10, *p*-value = 0.02).

**Table 1:** Changes in secondhand smoke (SHS) exposure before and after the implementation of smoke-free policies.

Dependent variable	Coefficient		SE	*t*-statistics	*P*-value	Adjusted *R*^2^	Durbin–Watson statistic
Log(SHS exposure in the workplace)	*β*_0_	1.56	0.10	14.38	1.77E-08***	0.88	1.00*
	*β*_1_	0.02	0.03	0.69	0.50		
	*β*_2_	0.18	0.09	1.99	0.07		
	*β*_3_	−0.10	0.04	−2.63	0.02*		
SHS exposure in the workplace	*β*_0_	37.00	6.63	5.58	0.00***	0.87	0.91*
	*β*_1_	2.61	2.42	1.07	0.30		
	*β*_2_	9.81	5.72	1.71	0.11		
	*β*_3_	−7.29	2.47	−2.94	0.01*		
Log(SHS exposure at home)	*β*_0_	1.26	0.06	19.40	7.39E-10***	0.94	1.39
	*β*_1_	−0.02	0.02	−1.08	0.29		
	*β*_2_	0.10	0.05	1.80	0.09		
	*β*_3_	−0.03	0.02	−1.56	0.14		
SHS exposure at home	*β*_0_	18.40	1.29	14.21	2E-08***	0.95	1.65
	*β*_1_	−1.00	0.47	−2.11	0.05		
	*β*_2_	0.96	1.11	0.85	0.40		
	*β*_3_	−0.15	0.48	−0.32	0.75		
Log(Primary cigarette smoking rate)	*β*_0_	1.53	0.01	77.69	<2E-16***	0.87	1.82
	*β*_1_	−0.02	0.005	−4.00	0.001**		
	*β*_2_	0.03	0.02	1.74	0.10		
	*β*_3_	0.00	0.00	1.52	0.15		
Primary cigarette smoking rate	*β*_0_	34.05	1.30	26.05	1.32E-12***	0.85	1.68
	*β*_1_	−1.42	0.33	−4.23	0.00***		
	*β*_2_	2.21	1.36	1.62	0.12		
	*β*_3_	0.76	0.36	2.10	0.05		

*p<0.05, **p<0.01, ***p<0.001.

**Figure 2:**
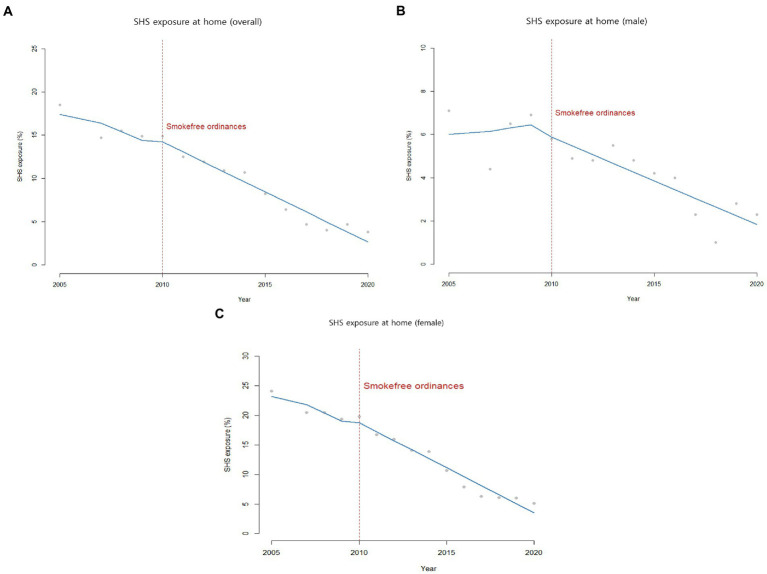
SHS exposure at home among adults in ROK (2005-2020).

Prior to the introduction of municipal ordinances, SHS exposure at home was already declining from 18.5% in 2005 to 14.9% in 2009. After the policy implementation, the decreasing trend continued from 14.9% in 2010 to 3.8% in 2020 without any intercept change (*β*_2_ = 0.10, *p*-value = 0.09) or slope gradient change (*β*_3_ = −0.03, *p*-value = 0.14) over time (Table 1; Figure 2).

**Figure 3:**
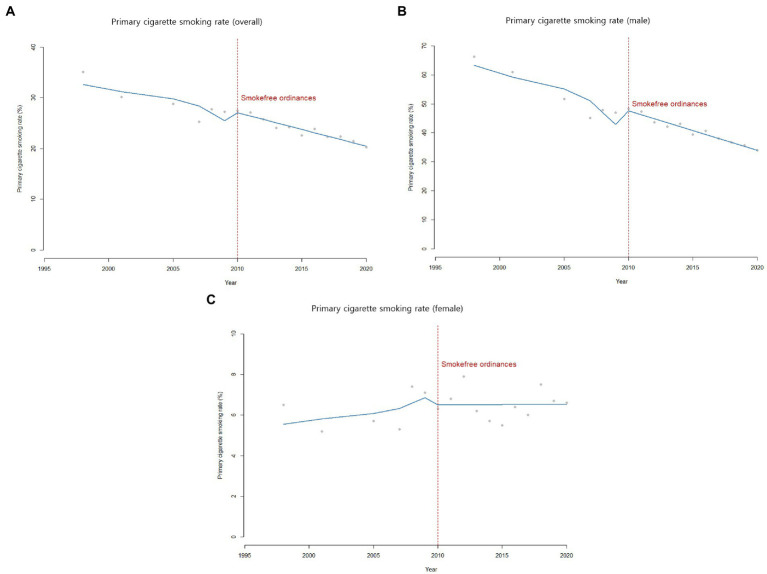
Primary cigarette smoking rate among adults in ROK (1998-2020).

The primary cigarette smoking rate showed a similar declining trend from 35.1% in 1998 to 27.3% in 2009, with no observed decline in the intercept (*β*_2_ = 0.03, *p*-value = 0.10) or the slope (*β*_3_ = 0.008, *p*-value = 0.15) after 2010. While the trend was somewhat stagnant around 2010, it continued to decrease from 27.5% in 2010 to 20.3% in 2020 (Table 1; Figure 3).

Even though overall downward trends observed in SHS exposure at home and primary cigarette smoking rate were significant, the policy implementation in 2010 did not result in any immediate level changes nor accelerated the overall trend.

### 3.2. Stratified analysis by sex

In stratified analysis by sex, the secular trend in SHS exposure in the workplace for both male and female showed similar changes, reaching a peak in 2010 and dropping from 44.6% in 2005 to 14.1% in 2020 among males and from 31.8% in 2005 to 6.6% in 2020 among females (Table 2; Figure 1).

**Table 2:** Subgroup analysis of secondhand smoke (SHS) exposure before and after the implementation of smoke-free policies by sex.

Dependent variable	Subgroup	Coefficient		SE	*t*-statistics	*P*-value	Adjusted *R*^2^	Durbin–Watson statistic
Log(SHS exposure in the workplace)	Male	*β*_0_	1.65	0.10	16.00	5.76E-09***	0.87	1.01*
		*β*_1_	0.02	0.03	0.57	0.57		
		*β*_2_	0.19	0.08	2.16	0.05		
		*β*_3_	−0.09	0.03	−2.47	0.03*		
	Female	*β*_0_	1.48	0.12	12.18	9.98E-08***	0.89	1.06*
		*β*_1_	0.03	0.04	0.68	0.50		
		*β*_2_	0.20	0.10	1.93	0.07		
		*β*_3_	−0.12	0.04	−2.65	0.02*		
SHS exposure in the workplace	Male	*β*_0_	45.55	7.55	6.03	8.53E-05***	0.87	0.95*
		*β*_1_	2.43	2.75	0.88	0.39		
		*β*_2_	13.55	6.52	2.08	0.06		
		*β*_3_	−7.86	2.81	−2.78	0.01*		
	Female	*β*_0_	30.25	5.83	5.18	0.00***	0.88	0.87*
		*β*_1_	2.49	2.14	1.16	0.27		
		*β*_2_	7.72	5.06	1.52	0.15		
		*β*_3_	−6.62	2.19	−3.02	0.01*		
Log(SHS exposure at home)	Male	*β*_0_	0.75	0.18	4.14	0.00**	0.57	1.97
		*β*_1_	0.01	0.06	0.19	0.84		
		*β*_2_	0.05	0.15	0.34	0.73		
		*β*_3_	−0.06	0.06	−0.98	0.34		
	Female	*β*_0_	1.39	0.05	27.10	2.01E-11***	0.96	1.17*
		*β*_1_	−0.02	0.01	−1.50	0.16		
		*β*_2_	0.09	0.04	2.20	0.04*		
		*β*_3_	−0.03	0.01	−1.80	0.09		
SHS exposure at home	Male	*β*_0_	5.85	1.17	4.98	0.00***	0.71	2.10
		*β*_1_	0.15	0.42	0.35	0.73		
		*β*_2_	−0.16	1.01	−0.16	0.87		
		*β*_3_	−0.55	0.43	−1.26	0.23		
	Female	*β*_0_	24.65	1.46	16.88	3.26E-9***	0.96	1.34*
		*β*_1_	−1.41	0.53	−2.64	0.02*		
		*β*_2_	1.27	1.26	1.01	0.33		
		*β*_3_	−0.11	0.54	−0.20	0.83		
Log(Primary cigarette smoking rate)	Male	*β*_0_	1.83	0.01	93.51	<2E-16***	0.92	1.69
		*β*_1_	−0.03	0.00	−6.36	2.46E-5***		
		*β*_2_	0.05	0.02	2.68	0.01*		
		*β*_3_	0.01	0.00	3.24	0.00**		
	Female	*β*_0_	0.72	0.05	14.43	2.22E-9***	−0.02	1.82
		*β*_1_	0.01	0.01	1.37	0.19		
		*β*_2_	−0.02	0.05	−0.43	0.66		
		*β*_3_	−0.01	0.01	−1.25	0.23		
Primary cigarette smoking rate	Male	*β*_0_	67.37	2.39	28.11	4.98E-13***	0.90	1.48*
		*β*_1_	−4.06	0.61	−6.61	1.68E-05***		
		*β*_2_	6.04	2.49	2.41	0.03*		
		*β*_3_	2.70	0.66	4.08	0.00**		
	Female	*β*_0_	5.28	0.74	7.09	8.17E0-6***	−0.03	1.84
		*β*_1_	0.26	0.19	1.37	0.19		
		*β*_2_	−0.35	0.77	−0.46	0.65		
		*β*_3_	−0.26	0.20	−1.26	0.22		

*p<0.05, **p<0.01, ***p<0.001.

SHS exposure at home for female continuously dropped from 24.1% in 2005 to 5.1% in 2020. Meanwhile, SHS exposure at home for male plateaued between 2005 and 2009, and then decreased from 7.1% in 2005 to 2.3% in 2020 (Table 2; Figure 2).

Primary cigarette smoking rate among female plateaued from 1998 to 2020 around 6 to 8%. Meanwhile, primary cigarette smoking rate among male continuously decreased over the same period (Table 2; Figure 3). Nevertheless, the primary cigarette smoking rate remains particularly high among males even now.

Online Supplementary Tables 1–3 provide characteristics of the study sample by each measure and Supplementary Tables 4, 5 show results of the additional analyses conducted for this study—for the years 2009 and 2011 (before and after the change in municipal ordinances). These analyses showed accelerated downward trends for SHS exposure in the workplace since the policy introduction in 2010.

## 4. Discussion

We found changes in SHS exposure in ROK after municipal ordinances under the National Health Promotion Act in 2010 strengthened local governments’ authority to designate smoke-free places and levy fines for noncompliance. Unfortunately, the legislation did not affect SHS exposure at home or the overall primary cigarette smoking rate. However, SHS exposure in the workplace did reverse course from an increasing trend (prior to 2010) to a decreasing trend after the adoption of the municipal ordinances in 2010. There were no sex differences with regard to this national policy’s effectiveness in changing the ongoing secular trends of SHS exposure in the workplace after 2010. No sex differences were seen in the primary cigarette smoking rate as well—i.e., in male (declining trend remains) and female (plateaued trend remains).

This study showed that strengthening the role of local governments and allowing them to charge penalties helped reduce SHS exposure in the workplace. It is well known that partial and sporadic smoking restrictions are not always enough to protect the public or workers from SHS exposure (28–30). This study’s results align with some of the findings described in previous studies. Park et al. (23) reported that the revision of the national smoke-free legislation in 2010 effectively reduced cotinine concentration in urine, a biomarker of exposure to tobacco, among non-smoking workers from 2009 to 2011 despite non-representative study population-based results (23). Ko (32) reported that the smoke-free legislation in ROK helped increase the number of quit attempts among smokers despite no changes in the primary cigarette smoking rate itself (32).

Some studies have raised concerns that restricting smoking in public places would shift the behavior to private spaces, e.g., homes and cars (33, 34). However, others have not found this to be the case. For example, Xiaohua et al. (35) reported no displacement effect on smoking venues in Guangzhou. Instead, they observed a significant decline in self-reported overall smoking behaviors at workplaces, restaurants, and hotels while the level remained high at home before and after the change in the smoke-free legislation(35). Similarly, in our data, we found no evidence of an increase in SHS exposure at home.

In our study, the exposure to SHS in the home decreased over time, without significant trend change before and after the policy intervention period. It showed a continued decline in the prevalence of smoking, an increase in the number of smokers who confine their smoking near home environment, and increased public awareness and compliance with smoke-free policies (36). Due to the strict local government policy to restrict smoking in indoor areas, smokers appear to have moved their smoking behaviors outdoor (on the streets, apartment verandas) and in apartment hallways, which have become the new major source of SHS exposure in ROK (37). Complete prohibition of smoking indoors is the only way to protect non-smokers from SHS exposure (38), since there is a substitution effect from smoking ban areas to less restricted areas. Devising strategies to expand policies to restrict SHS exposure beyond banning smoking in public places would be the next policy target.

This study has several limitations that should be considered when interpreting the results. First, the effects of the national legislation on SHS exposure at the workplace did not include all public places due to limitations with accessing relevant data. As a result, we only looked at results from the workplace and at home for the sampled period. SHS exposure data became more comprehensive in 2013 as the Korean government began collecting these data; however, the study’s intervention period was prior to this year. Second, this study could not consider the impact of significant increase in tobacco tax on SHS exposure due to non-availability of tobacco consumption amount for the study period while significant increase in tobacco tax is well known as one of the most effective measure for reducing tobacco use (39). Previous studies conducted in ROK showed that the increased tobacco taxation (approximately 2 USD) in 2015 had reduced tobacco consumption amount in short-term and long-term periods (40, 41). As reduced amount of tobacco consumption might have affected SHS exposure among the non-smokers, our study results (impact of the municipal smoke-free ordinances) might have been overestimated due to the overlapped tobacco taxation policy impact since 2015. Third, there might have been a possible measurement error in SHS exposure, which was assessed by self-report. While there are many types of smoked tobacco products such as electronic cigarettes, heat-not-burn tobacco products, or vape products these days, the SHS variables only target non-smokers’ exposure to tobacco smoke. Also, SHS exposure assessed by self-report alone may not reflect the actual SHS exposure level as biomarkers such as urine concentration was not included in the analysis (42). Fourth, causal inferences cannot be made from this study because the results are based on aggregated data with no specific control group (43). Fifth, since only annually aggregated data at the national level were available, municipality-level sub-data analysis and adjustment for seasonality were not possible.

In conclusion, this study found that the role of national legislation enabling local governments to designate smoke-free places and establish penalty fines was instrumental in reducing SHS exposure in the workplace. However, direct effects on SHS exposure at home were not demonstrated and could not be estimated in the study. Further research on how to reduce tobacco smoke beyond restrictions in public places should be conducted to help inform and guide tobacco control policies, so that innocent non-smokers can be better protected from unwanted exposure to tobacco smoke. As tobacco use is a learned and socially mediated behavior (44), the primary purpose of tobacco control policies should be to change the social norms in the environment (45). Other complementary approaches, such as counter-messages, community engagement, and taxation strategy, would be also necessary to amplify the changes in social norms and behavior, thereby reducing prevalence and protecting non-smokers in society.

## Data availability statement

Publicly available datasets were analyzed in this study. This data can be found at: https://knhanes.kdca.go.kr/knhanes/sub03/sub03_02_05.do.

## Author contributions

SK conceived the study and analyzed and drafted the manuscript. SK and JO designed the study and interpreted the results. YL and CH contributed to the interpretation of results and revised and reviewed the manuscript for intellectual content. MK revised and reviewed the manuscript. IK and JO provided feedback and helped revise the manuscript throughout the process. All authors contributed to the article and approved the submitted version.

## Conflict of interest

The authors declare that the research was conducted in the absence of any commercial or financial conflicts.

## Publisher’s note

All claims expressed in this article are solely those of the authors and do not necessarily represent those of their affiliated organizations, or those of the publisher, the editors and the reviewers. Any product that may be evaluated in this article, or claim that may be made by its manufacturer, is not guaranteed or endorsed by the publisher.

## Footnotes

^1^KNHANES is composed of three surveys: health interview, health examination, and nutrition survey. In this study, we used health interview data only. Target population of KNHANES’ health interview survey comprises non-institutionalized Korean citizens residing in Korea aged 19 years and above. The sampling plan follows a multi-stage clustered probability design. For example, in the 2011 survey, 192 primary sampling units (PSUs) were drawn from approximately 200,000 geographically defined PSUs for the whole country. A PSU consisted of an average of 60 households, and 20 final target households were sampled for each PSU using systematic sampling (Kweon et al., 2014 [26]). Where non-response rates are not provided for each question, the overall missingness for KNHNES items ranges between 0.8% and 1.8%. Non-response weight adjustment was used in the case of unit non-response; item non-response was considered negligible.
