# Supplementary material for: Effects of municipal smoke-free ordinances on secondhand smoke exposure in the Republic of Korea
Source: Front Public Health. 2023 Mar 27;11:1062753. doi: 10.3389/fpubh.2023.1062753 (PMC10084937; doi:10.3389/fpubh.2023.1062753)
Supplement: Supplementary file 1 [file Table_1.DOCX]

Supplementary Material

Supplementary Table 1. Characteristics of the study sample for SHS exposure at home

| Variables | | Participants (%) | | | | | | | |
| --- | --- | --- | --- | --- | --- | --- | --- | --- | --- |
|  |  | 2005 | 2007 | 2008 | 2009 | 2010 | 2011 | 2012 | 2013 |
| SHS exposure at home (overall, standardized) | | 5,747 (18.5) | 2,362 (14.7) | 5,269 (15.5) | 5,805 (14.9) | 4,917 (14.9) | 4,790 (12.5) | 4,537 (11.9) | 4,304 (10.9) |
| SHS exposure at home (male, standardized) | | 1,695 (7.1) | 719 (4.4) | 1,57 (6.5) | 1,834 (6.9) | 1,547 (5.8) | 1,506 (4.9) | 1,427 (4.8) | 1,378 (5.5) |
| SHS exposure at home (female, standardized) | | 4,052 (24.1) | 1,643 (20.5) | 3,698 (20.5) | 3,971 (19.4) | 3,370 (19.8) | 3,284 (16.7) | 3,110 (16.0) | 2,926 (14.1) |
| Age | 19-64 | 4,853 (19.5) | 1,755 (15.1) | 3,990 (15.8) | 4,375 (15.4) | 3,724 (15.7) | 3,526 (12.7) | 3,268 (12.4) | 3,240 (11.5) |
|  | 65 over | 894 (10.7) | 607 (10.3) | 1,279 (12.1) | 1,430 (10.0) | 1,193 (7.4) | 1,264 (8.2) | 1,269 (5.8) | 1,064 (6.5) |
| Region | Urban | 4,611 (18.1) | 1,672 (14.7) | 3,839 (14.5) | 4,295 (14.7) | 3,840 (14.2) | 3,846 (11.8) | 3,617 (11.6) | 3,488 (10.6) |
|  | Rural | 1,136 (19.2) | 690 (13.3) | 1,430 (18.4) | 1,510 (13.6) | 1,077 (15.0) | 944 (12.6) | 920 (9.9) | 816 (10.8) |
| Income | 1Q | 1,104 (19.0) | 413 (21.7) | 971 (19.3) | 1,073 (14.9) | 903 (15.6) | 887 (12.1) | 821 (13.2) | 791 (10.1) |
|  | 2Q | 1,107 (19.6) | 445 (12.8) | 994 (18.2) | 1,154 (15.1) | 953 (16.0) | 918 (11.1) | 884 (10.1) | 826 (12.6) |
|  | 3Q | 1,123 (21.3) | 450 (18.9) | 1,015 (15.0) | 1,143 (15.6) | 1 012 (12.9) | 959 (12.6) | 898 (11.7) | 867 (11.0) |
|  | 3Q | 1,175 (17.8) | 457 (11.0) | 1,066 (11.5) | 1,191 (16.0) | 986 (12.4) | 979 (14.1) | 934 (9.3) | 887 (9.4) |
|  | 4Q | 1,179 (13.7) | 484 (9.4) | 1,056 (12.3) | 1,188 (10.4) | 999 (14.6) | 1,005 (9.8) | 950 (11.7) | 912 (10.2) |

Supplementary Table 1. Characteristics of the study sample for SHS exposure at home (continued)

| Variables | | Participants (%) | | | | | | |
| --- | --- | --- | --- | --- | --- | --- | --- | --- |
|  |  | 2014 | 2015 | 2016 | 2017 | 2018 | 2019 | 2020 |
| SHS exposure at home (overall, standardized) | | 4,208 (10.7) | 4,456 (8.2) | 4,890 (6.4) | 5,023 (4.7) | 5,062 (4.0) | 5,110 (4.7) | 4,884 (3.9) |
| SHS exposure at home (male, standardized) | | 1,307 (4.8) | 1,542 (4.2) | 1,652 (4.0) | 1,773 (2.3) | 1,794 (1.0) | 1,846 (2.8) | 1,819 (2.3) |
| SHS exposure at home (female, standardized) | | 2,901 (13.9) | 2,914 (10.7) | 3,238 (7.9) | 3,250 (6.3) | 3,268 (6.1) | 3,264 (6.0) | 3,065 (5.1) |
| Age | 19-64 | 3,018 (11.2) | 3,212 (8.8) | 3,510 (6.7) | 3,592 (4.9) | 3,604 (4.3) | 3,618 (4.8) | 3,391 (4.1) |
|  | 65 over | 1,190 (6.2) | 1,244 (5.0) | 1,380 (3.5) | 1,431 (3.9) | 1,458 (2.2) | 1,49 (2.7) | 1,493 (2.6) |
| Region | Urban | 3,403 (10.4) | 3,589 (8.2) | 3,956 (5.6) | 4,110 (4.2) | 4,165 (3.8) | 4,089 (4.4) | 3,881 (3.9) |
|  | Rural | 805 (9.8) | 867 (8.0) | 934 (8.7) | 913 (7.3) | 897 (4.2) | 1,021 (4.6) | 1,003 (3.2) |
| Income | 1Q | 768 (12.2) | 831 (8.4) | 934 (7.6) | 935 (6.7) | 954 (5.9) | 939 (4.5) | 915 (3.9) |
|  | 2Q | 828 (9.0) | 890 (8.5) | 945 (6.2) | 981 (4.7) | 985 (4.2) | 1,003 (5.2) | 943 (4.4) |
|  | 3Q | 836 (9.8) | 889 (9.0) | 988 (6.8) | 1,000 (5.7) | 1,018 (3.1) | 1,009 (4.5) | 984 (4.3) |
|  | 4Q | 865 (11.1) | 895 (7.8) | 995 (5.4) | 1,030 (3.7) | 1,029 (3.5) | 1,050 (4.3) | 1,008 (3.4) |
|  | 5Q | 894 (9.4) | 927 (7.5) | 1,014 (4.4) | 1,061 (2.8) | 1,061 (2.8) | 1,085 (3.8) | 1,016 (2.8) |

Supplementary Table 2. Characteristics of the study sample for SHS exposure in the workplace

| Variables | | Participants (%) | | | | | | | |
| --- | --- | --- | --- | --- | --- | --- | --- | --- | --- |
|  |  | 2005 | 2007 | 2008 | 2009 | 2010 | 2011 | 2012 | 2013 |
| SHS exposure in the workplace  (overall, standardized) | | 3,182 (63.9) | 1,106 (46.0) | 2,838 (45.4) | 3,169 (45.8) | 2,703 (49.2) | 2,591 (45.2) | 2,463 (46.1) | 2,302 (47.4) |
| SHS exposure in the workplace  (male, standardized) | | 1,251 (44.6) | 460 (55.2) | 1,118 (53.4) | 1,273 (53.3) | 1,108 (58.7) | 1,065 (55.3) | 993 (54.5) | 924 (57.3) |
| SHS exposure in the workplace  (female, standardized) | | 1,931 (31.8) | 646 (36.4) | 1,720 (38.2) | 1,896 (39.5) | 1,595 (41.8) | 1,526 (37.2) | 1,470 (38.9) | 1,378 (38.7) |
| Age | 19-64 | 2,923 (40.5) | 959 (48.0) | 2,413 (47.3) | 2,690 (48.7) | 2,319 (51.5) | 2,161 (46.7) | 2,056 (48.0) | 2,003 (49.7) |
|  | 65 over | 259 (12.5) | 147 (27.1) | 425 (24.3) | 479 (19.5) | 384 (23.6) | 430 (21.5) | 407 (16.7) | 299 (33.1) |
| Region | Urban | 2,471 (42.2) | 750 (47.0) | 1,889 (47.6) | 2,181 (48.2) | 1,986 (50.2) | 1,985 (45.5) | 1,897 (46.4) | 1,811 (49.0) |
|  | Rural | 711 (24.8) | 356 (46.0) | 949 (38.2) | 988 (40.1) | 717 (45.9) | 606 (39.8) | 566 (40.7) | 491 (45.5) |
| Income | 1Q | 498 (35.5) | 200 (42.8) | 515 (44.8) | 539 (46.8) | 435 (49.5) | 430 (42.8) | 395 (43.7) | 350 (46.7) |
|  | 2Q | 592 (35.8) | 209 (50.5) | 546 (48.7) | 624 (46.9) | 520 (45.4) | 488 (41.0) | 487 (40.1) | 432 (53.7) |
|  | 3Q | 641 (41.0) | 214 (55.1) | 570 (43.4) | 650 (49.4) | 609 (50.0) | 537 (45.4) | 500 (50.1) | 496 (42.9) |
|  | 4Q | 702 (40.8) | 223 (44.6) | 595 (46.8) | 667 (46.0) | 544 (45.3) | 560 (46.3) | 524 (44.3) | 520 (50.4) |
|  | 5Q | 723 (37.6) | 234 (43.5) | 554 (45.0) | 667 (43.7) | 566 (55.6) | 558 (46.0) | 536 (48.3) | 494 (47.7) |

Supplementary Table 2. Characteristics of the study sample for SHS exposure in the workplace (continued)

| Variables | | Participants (%) | | | | | | |
| --- | --- | --- | --- | --- | --- | --- | --- | --- |
|  |  | 2014 | 2015 | 2016 | 2017 | 2018 | 2019 | 2020 |
| SHS exposure in the workplace  (overall, standardized) | | 2,148 (40.1) | 2,343 (26.9) | 2,627(17.4) | 2,756 (12.7) | 2,887 (11.5) | 2,844 (14.1) | 2,598 (10.3) |
| SHS exposure in the workplace  (male, standardized) | | 829 (49.1) | 982 (36.2) | 1,097 (23.5) | 1,142 (17.3) | 1,187 (14.4) | 1,231 (18.1) | 1,124 (14.1) |
| SHS exposure in the workplace  (female, standardized) | | 1,319 (32.8) | 1,361 (18.5) | 1,530 (12.1) | 1,614 (8.5) | 1,700 (8.7) | 1,613 (10.3) | 1,474 (6.6) |
| Age | 19-64 | 1,789 (42.3) | 1,970 (28.9) | 2,200 (18.7) | 2,336 (14.1) | 2,420 (12.5) | 2,375 (15.1) | 2,140 (11.2) |
|  | 65 over | 359 (24.2) | 373 (15.2) | 427 (9.2) | 420 (7.0) | 467 (6.0) | 469 (7.3) | 458 (6.7) |
| Region | Urban | 1,706 (42.0) | 1,841 (26.3) | 2,087 (17.0) | 2,222 (13.5) | 2,380 (11.7) | 2,261 (14.9) | 2,066 (10.8) |
|  | Rural | 422 (34.7) | 502 (34.0) | 540 (21.9) | 534 (12.9) | 507 (13.0) | 583 (10.8) | 532 (9.9) |
| Income | 1Q | 330 (46.7) | 344 (32.1) | 409 (23.2) | 410 (13.2) | 442 (18.5) | 403 (17.6) | 372 (12.9) |
|  | 2Q | 438 (34.6) | 465 (25.7) | 504 (18.3) | 558 (17.0) | 555 (13.4) | 536 (19.3) | 497 (11.8) |
|  | 3Q | 437 (41.6) | 505 (28.6) | 596 (14.9) | 571 (15.4) | 572 (12.9) | 589 (12.5) | 553 (10.1) |
|  | 4Q | 449 (40.6) | 511 (26.8) | 545 (18.1) | 599 (12.0) | 643 (10.4) | 647 (10.2) | 601 (12.4) |
|  | 5Q | 488 (41.1) | 509 (26.0) | 570 (15.7) | 617 (10.0) | 669 (6.0) | 663 (13.0) | 572 (7.1) |

Supplementary Table 3. Characteristics of the study sample for primary cigarette smoking rate

| Variables | | Participants (%) | | | | | | | |
| --- | --- | --- | --- | --- | --- | --- | --- | --- | --- |
|  |  | 1998 | 2001 | 2005 | 2007 | 2008 | 2009 | 2010 | 2011 |
| Primary cigarette smoking rate  (overall, standardized) | | 8,823 (35.1) | 8,069 (30.2) | 7,801 (28.8) | 2,980 (25.3) | 6,797 (27.8) | 7,470 (27.3) | 6,256 (27.5) | 6,023 (27.1) |
| Primary cigarette smoking rate  (male, standardized) | | 4,110 (66.3) | 3,677 (60.9) | 3,509 (51.7) | 1,241 (45.1) | 2,838 (47.8) | 3,234 (47.0) | 2,704 (48.3) | 2,557 (47.3) |
| Primary cigarette smoking rate  (female, standardized) | | 4,713 (6.5) | 4,392 (5.2) | 4,292 (5.7) | 1,739 (5.3) | 3,959 (7.4) | 4,236 (7.1) | 3,552 (6.3) | 3,466 (6.8) |
| Age | 19-64 | 7,779 (36.0) | 7,091 (30.9) | 6,704 (30.3) | 2,259 (26.5) | 5,286 (28.9) | 5,816 (28.6) | 4,882 (29.1) | 4,559 (28.3) |
|  | 65 over | 1,044 (28.7) | 978 (24.7) | 1,097 (17.4) | 721 (14.7) | 1,511 (16.6) | 1,654 (14.0) | 1,374 (13.0) | 1,464 (14.2) |
| Region | Urban | 5,716 (35.0) | 6,417 (29.6) | 6,237 (28.5) | 2,117 (25.2) | 4,999 (27.8) | 5,553 (26.8) | 4,890 (27.0) | 4,809 (25.9) |
|  | Rural | 3,107 (36.1) | 1,652 (32.7) | 1,564 (30.2) | 863 (24.2) | 1,798 (25.1) | 1,917 (26.0) | 1,366 (26.6) | 1,214 (28.1) |
| Income | 1Q | 1,737 (38.5) | 1,335 (34.7) | 1,599 (33.2) | 561 (31.2) | 1,323 (32.5) | 1,466 (32.0) | 1,215 (30.9) | 1,204 (32.4) |
|  | 2Q | 1,721 (37.0) | 1,544 (31.3) | 1,549 (30.7) | 570 (26.7) | 1,328 (28.3) | 1,506 (28.4) | 1,248 (28.6) | 1,184 (28.0) |
|  | 3Q | 1,788 (34.9) | 1,463 (30.3) | 1,520 (28.8) | 567 (24.6) | 1,296 (27.0) | 1,475 (26.6) | 1,271 (25.0) | 1,190 (25.2) |
|  | 4Q | 1,815 (33.9) | 1,531 (27.3) | 1,523 (25.5) | 571 (24.6) | 1,341 (26.5) | 1,490 (24.7) | 1,214 (25.3) | 1,191 (22.2) |
|  | 5Q | 1,762 (32.7) | 1,726 (28.1) | 1,523 (25.4) | 573 (18.8) | 1,296 (22.5) | 1,452 (20.8) | 1,228 (24.0) | 1,206 (22.7) |

Supplementary Table 3. Characteristics of the study sample for primary cigarette smoking rate (continued)

| Variables | | Participants (%) | | | | | | | | |
| --- | --- | --- | --- | --- | --- | --- | --- | --- | --- | --- |
|  |  | 2012 | 2013 | 2014 | 2015 | 2016 | 2017 | 2018 | 2019 | 2020 |
| Primary cigarette smoking rate  (overall, standardized) | | 5,591 (25.8) | 5,338 (24.1) | 5,192 (24.2) | 5,405 (22.6) | 6,015 (23.9) | 6,115 (22.3) | 6,183 (22.4) | 6,190 (21.5) | 5,858 (20.6) |
| Primary cigarette smoking rate  (male, standardized) | | 2,309 (43.7) | 2,254 (42.2) | 2,154 (43.2) | 2,352 (39.4) | 2,592 (40.7) | 2,717 (38.1) | 2,713 (36.7) | 2,744 (35.7) | 2,627 (34.0) |
| Primary cigarette smoking rate  (female, standardized) | | 3,282 (7.9) | 3,084 (6.2) | 3,038 (5.7) | 3,053 (5.5) | 3,423 (6.4) | 3,398 (6.0) | 3,470 (7.5) | 3,446 (6.7) | 3,231 (6.6) |
| Age | 19-64 | 4,161 (27.2) | 4,137 (25.3) | 3,851 (25.4) | 4,022 (23.7) | 4,489 (25.1) | 4,528 (23.3) | 4,595 (23.8) | 4,537 (22.3) | 4,217 (21.4) |
|  | 65 over | 1,430 (11.7) | 1,201 (11.1) | 1,341 (11.1) | 1,383 (9.8) | 1,526 (9.7) | 1,587 (9.6) | 1,588 (8.2) | 1,653 (10.3) | 1,641 (9.2) |
| Region | Urban | 4,466 (25.1) | 4,307 (22.7) | 4,192 (22.7) | 4,364 (21.7) | 4,853 (22.1) | 4,991 (20.6) | 5,060 (20.7) | 4,958 (20.3) | 4,661 (19.5) |
|  | Rural | 1,125 (24.6) | 1,031 (25.4) | 1,000 (26.0) | 1,041 (21.0) | 1,162 (25.2) | 1,124 (23.7) | 1,123 (23.8) | 1,232 (19.6) | 1,197 (17.8) |
| Income | 1Q | 1,079 (29.2) | 1,036 (27.6) | 1,010 (27.7) | 1,045 (25.2) | 1,192 (25.0) | 1,211 (24.9) | 1,234 (24.5) | 1,236 (26.9) | 1,149 (22.5) |
|  | 2Q | 1,116 (27.6) | 1,052 (26.0) | 1,037 (23.6) | 1,076 (20.8) | 1,201 (25.1) | 1,215 (23.9) | 1,236 (25.0) | 1,222 (20.0) | 1,162 (22.4) |
|  | 3Q | 1,087 (22.6) | 1,080 (23.3) | 1,036 (24.2) | 1,081 (23.1) | 1,201 (21.5) | 1,221 (21.5) | 1,242 (20.6) | 1,225 (22.4) | 1,172 (18.4) |
|  | 4Q | 1,110 (21.6) | 1,063 (20.0) | 1,051 (22.3) | 1,084 (20.5) | 1,203 (21.8) | 1,228 (20.0) | 1,222 (19.2) | 1,244 (18.6) | 1,176 (17.3) |
|  | 5Q | 1,134 (22.6) | 1,079 (19.1) | 1,039 (18.6) | 1,088 (18.0) | 1,199 (19.6) | 1,220 (15.1) | 1,231 (15.8) | 1,236 (13.7) | 1,179 (16.0) |

Supplementary Table 4. Changes in secondhand smoke exposure before and after the year 2009

| Dependent variable | Coefficient | | SE | t-statistics | P value | Adjusted R^2^ | Durbin-Watson statistic |
| --- | --- | --- | --- | --- | --- | --- | --- |
| log(SHS exposure in the workplace) | β_0_ | 1.53 | 0.15 | 9.81 | 8.87E-07*** | 0.84 | 0.91 |
|  | β_1_ | 0.04 | 0.07 | 0.62 | 0.54 |  |  |
|  | β_2_ | 0.20 | 0.11 | 1.79 | 0.09 |  |  |
|  | β_3_ | -0.11 | 0.07 | -1.59 | 0.13 |  |  |
| SHS exposure in the workplace | β_0_ | 34.26 | 9.27 | 3.69 | 0.00** | 0.84 | 0.93 |
|  | β_1_ | 4.25 | 4.29 | 0.99 | 0.34 |  |  |
|  | β_2_ | 11.09 | 6.68 | 1.66 | 0.12 |  |  |
|  | β_3_ | -8.49 | 4.32 | -1.96 | 0.07 |  |  |
| log(SHS exposure at home) | β_0_ | 1.28 | 0.09 | 14.01 | 6.69E-08*** | 0.92 | 1.42 |
|  | β_1_ | -0.03 | 0.04 | -0.90 | 0.38 |  |  |
|  | β_2_ | 0.12 | 0.06 | 1.92 | 0.08 |  |  |
|  | β_3_ | -0.02 | 0.04 | -0.53 | 0.60 |  |  |
| SHS exposure at home | β_0_ | 19.23 | 1.52 | 12.64 | 1.78E-07*** | 0.95 | 2.28 |
|  | β_1_ | -1.50 | 0.70 | -2.13 | 0.05 |  |  |
|  | β_2_ | 1.88 | 1.11 | 1.69 | 0.12 |  |  |
|  | β_3_ | 0.30 | 0.71 | 0.42 | 0.67 |  |  |
| log(Primary cigarette smoking rate) | β_0_ | 1.55 | 0.01 | 79.07 | <2E-16*** | 0.90 | 2.61 |
|  | β_1_ | -0.02 | 0.00 | -4.72 | 0.00*** |  |  |
|  | β_2_ | 0.04 | 0.01 | 2.41 | 0.03* |  |  |
|  | β_3_ | 0.01 | 0.00 | 2.67 | 0.01* |  |  |
| Primary cigarette smoking rate | β_0_ | 35.29 | 1.27 | 27.67 | 6.1E-13*** | 0.89 | 2.45 |
|  | β_1_ | -1.95 | 0.38 | -5.07 | 0.00*** |  |  |
|  | β_2_ | 2.71 | 1.20 | 2.25 | 0.04* |  |  |
|  | β_3_ | 1.30 | 0.39 | 3.29 | 0.005** |  |  |

Supplementary Table 5. Changes in secondhand smoke exposure before and after the year 2011

| Dependent variable | Coefficient | | SE | t-statistics | P value | Adjusted R^2^ | Durbin-Watson statistic |
| --- | --- | --- | --- | --- | --- | --- | --- |
| log(SHS exposure in the workplace) | β_0_ | 1.57 | 0.08 | 18.29 | 1.39E-09*** | 0.90 | 1.18 |
|  | β_1_ | 0.02 | 0.02 | 0.95 | 0.36 |  |  |
|  | β_2_ | 0.13 | 0.08 | 1.57 | 0.14 |  |  |
|  | β_3_ | -0.11 | 0.02 | -4.02 | 0.00** |  |  |
| SHS exposure in the workplace | β_0_ | 37.34 | 5.53 | 6.75 | 3.15E-05*** | 0.88 | 1.01 |
|  | β_1_ | 2.44 | 1.66 | 1.46 | 0.17 |  |  |
|  | β_2_ | 4.60 | 5.44 | 095 | 0.41 |  |  |
|  | β_3_ | -7.34 | 1.76 | -4.15 | 0.00** |  |  |
| log(SHS exposure at home) | β_0_ | 1.24 | 0.05 | 21.84 | 9.05E-10*** | 0.93 | 1.59 |
|  | β_1_ | -0.01 | 0.01 | -1.05 | 0.31 |  |  |
|  | β_2_ | 0.06 | 0.05 | 1.03 | 0.32 |  |  |
|  | β_3_ | -0.04 | 0.01 | -2.66 | 0.02* |  |  |
| SHS exposure at home | β_0_ | 17.80 | 1.09 | 16.24 | 1.62E-08*** | 0.94 | 1.93 |
|  | β_1_ | -0.70 | 0.33 | -2.11 | 0.06 |  |  |
|  | β_2_ | -0.11 | 1.10 | -0.10 | 0.92 |  |  |
|  | β_3_ | -0.49 | 0.35 | -1.38 | 0.19 |  |  |
| log(Primary cigarette smoking rate) | β_0_ | 1.51 | 0.01 | 79.97 | <2E-16*** | 0.84 | 1.54 |
|  | β_1_ | -0.01 | 0.00 | -3.40 | 0.00** |  |  |
|  | β_2_ | 0.01 | 0.02 | 0.85 | 0.40 |  |  |
|  | β_3_ | 0.00 | 0.00 | 0.66 | 0.52 |  |  |
| Primary cigarette smoking rate | β_0_ | 33.08 | 1.31 | 25.14 | 2.08E-12*** | 0.82 | 1.42 |
|  | β_1_ | -1.05 | 0.29 | -3.59 | 0.00** |  |  |
|  | β_2_ | 1.20 | 1.50 | 0.80 | 0.43 |  |  |
|  | β_3_ | 0.42 | 0.34 | 1.25 | 0.23 |  |  |
